# Corticosteroid administration and glycemic outcomes during treatment of acute exacerbation of chronic obstructive pulmonary disease

**DOI:** 10.1016/j.ajmo.2022.100027

**Published:** 2022-11-07

**Authors:** Herman Joseph Johannesmeyer, Kayvan Moussavi, Kerry Anne Rambaran, Kristica Kolyouthapong

**Affiliations:** a Assistant Professor of Pharmacy Practice, Marshall B. Ketchum University, College of Pharmacy, 2575 Yorba Linda Blvd., Fullerton, CA 92831, United States; b Assistant Professor of Pharmacy Practice, Marshall B. Ketchum University, College of Pharmacy, United States; c Emergency Medicine Pharmacy Specialist, Scripps Mercy Hospital San Diego, United States; d Medication Safety Pharmacist, Mission Hospital – Mission Viejo, United States

**Keywords:** Corticosteroids, Chronic obstructive pulmonary disease, Acute exacerbation of chronic obstructive pulmonary disease, Hyperglycemia, Length of stay

## Abstract

**Background:**

While international guidelines recommend low doses of systemic corticosteroids for acute exacerbations of chronic obstructive pulmonary disease (AECOPD) clinical practice patterns show significant heterogeneity. Increasing doses of corticosteroids have inconsistently been associated with a greater risk of hyperglycemia.

**Methods:**

Patients admitted to inpatient services for AECOPD were retrospectively enrolled. Hospitalization corticosteroid doses, daily blood glucose levels, and other markers of corticosteroid excess were collected. Correlative and regression analyses were conducted to assess the relationship between corticosteroid dose and average hospitalization blood glucose.

**Results:**

Daily corticosteroid dose significantly predicted a higher blood glucose (rs=0.179, p=0.0095; p<0.0028 respectively) and cumulative corticosteroid dose predicted a longer hospital length of stay in bivariate and multivariate analyses (rs=0.679, p<0.0001; p<0.0001 respectively). Patients that experienced hypernatremia, hypokalemia, acute hyperglycemia, and acute hypertension received larger corticosteroid doses than patients that did not experience these complicating events.

**Conclusions:**

We identified that increasing amounts of corticosteroids administered to inpatients experiencing AECOPD experienced higher average hospitalization blood glucose values, protracted hospitalizations, and other untoward effects.

## Highlights

• International guidelines currently endorse low doses of corticosteroids for AECOPD.
• Higher daily corticosteroid doses were associated with higher average hospitalization blood glucose.
• Higher cumulative corticosteroid doses correlated with longer hospitalizations.

## Background

Chronic obstructive pulmonary disease (COPD) is defined as a limitation in airflow as a result of lung parenchymal damage and airway disease.^1^ COPD is one of the leading causes of mortality worldwide and is associated with an economic burden of over $50 billion in healthcare costs.^2^^,^^3^ Acute exacerbations of COPD (AECOPD) are particularly associated with significant short-term mortality, disease progression, and account for greater than 50% of the healthcare costs associated with COPD.^3^ Acute exacerbations have been shown to worsen patient quality of life both acutely and long-term after acute recovery.^4^, ^5^, ^6^ Risk of in-hospital mortality during an AECOPD is approximately 5% depending on the severity of the exacerbation.^7^^,^^8^

Recommended pharmacotherapy of AECOPD includes inhaled bronchodilators, judicious antibiotics, and systemic corticosteroids.^1^ In the 1999 SCCOPE trial the use of corticosteroids against placebo was shown to decrease the risk of the primary composite endpoint of treatment failure and was associated with shortened hospital length of stay in patients with acute COPD.^9^ Current treatment guidelines from the Global Initiative for Chronic Obstructive Lung Disease (GOLD) recommend prednisone 40 mg daily for five days; however, the optimal dose of corticosteroids has not been absolutely defined.^1^ This dosing recommendation was made in part from the results of the REDUCE Trial which showed lower cumulative systemic corticosteroid doses lead to non-inferior clinical outcomes relative to larger doses.^10^ In clinical practice corticosteroid dose for AECOPD varies widely between medical providers and is frequently adjusted to meet therapeutic goals.^11^^,^^12^

Hyperglycemia is a known and well-described adverse effect of corticosteroids.^13^^,^^14^ Short-term hyperglycemia has been shown to induce immune system dysregulation and, in inpatients, has been associated with poorer clinical outcomes including a protracted length of stay and in-hospital mortality.^15^^,^^16^ However, recent trials have noted an inconsistent relationship between corticosteroid dose and incidence of hyperglycemia in the treatment of AECOPD.^10^^,^^17^^,^^18^ The inconsistency of this relationship may be due in part to limitations in power, pre-existing medical conditions, the listing of hyperglycemia as an exploratory outcome of the study, or the statistical methods used within these studies treating hyperglycemia as a nominal outcome above or below a certain threshold as opposed to a continuous correlation.

With the addition of COPD as an item of the Centers for Medicare and Medicaid Services Hospital Readmission Reduction Program and recent proposals to add hyperglycemia as a marker of inpatient quality of care, hospitals now have additional financial incentive to reduce the risk of hyperglycemia during AECOPD treatment and subsequent readmissions of inpatients treated for AECOPD.^19^ The purpose of this study was to determine whether a correlation exists between corticosteroid dose in AECOPD and a patient's serum glucose during hospitalization. Due to positive outcomes in some related trials and biologic plausibility we hypothesized that in patients being treated for AECOPD increasing doses of corticosteroids would increase patient average hospitalization blood glucose.

## Methods

### Study design and enrollment

This study was a retrospective chart review of patients admitted with an AECOPD to Mission Hospital in Mission Viejo, California, United States of America, a 504 bed tertiary care community hospital. Screening for eligible patients was conducted by reviewing admitted inpatients between January 1^st^ to August 31^st^ 2020 with an International Classification of Disease – 10 (ICD-10) code indicating an AECOPD (code J44.1). Patients were included if they received two or more doses of corticosteroids during their hospitalization. Patients maintained on chronic outpatient corticosteroids were eligible for inclusion if they received two or more corticosteroid doses that represented a dose increase from their outpatient dose. Patients were excluded if they transferred to the hospital from an outside hospital. The institutional review board declared this study exempt from review.

### Data collection and outcomes

The primary objective of this study was to examine the effect of corticosteroid dose on serum glucose. Secondary objectives were to determine the relationship, if any, between corticosteroid dose on hospital length of stay and 30-day readmission and occurrence of corticosteroid-related side effects. Length of stay was calculated as the difference between the date and time of a patient's presentation to the hospital and their discharge from the hospital. Corticosteroid-related side effects were defined as hypokalemia (serum potassium < 3.5mEq/L), hypernatremia (serum sodium >145 mEq/L), acute hyperglycemia (serum glucose > 180 mg/dL [10 mmol/L]), and acute hypertension (systolic blood pressure above 180 mmHg or requirement of oral or intravenous as needed anti-hypertensives) from the point of initial systemic corticosteroid administration until hospital discharge.

Data was collected by manual data extraction from the patient's electronic medical record by HJ. Initial serum glucose was defined as the last serum glucose collected prior to administration of corticosteroids. Subsequent serum glucose values were morning whole blood measurements if available or, if unavailable, morning point of care glucose values. Average hospitalization serum glucose values were calculated on an individual patient basis as a mean of all of these values. Cumulative hospitalization corticosteroid dose was calculated as the sum of all corticosteroid medications converted to prednisone milligram equivalents. Conversions were made under the assumption that 5 mg of prednisone, 4 mg of methylprednisolone, and 0.75 mg of dexamethasone exerted equivalent corticosteroid effects.^20^

The acute hyperglycemia nominal outcome included all blood glucose readings, both whole blood and point of care testing. Simplified acute physiology score II (SAPS II) and BAP-65 were assessed at the time of initial respiratory failure identification.^21^^,^^22^ The SAPS II and BAP-65 are clinical acuity markers that utilize a variety of patient specific parameters to compute a risk of mortality. The SAPS II and BAP-65 are scored on a scale from 0 to 160 and Class I to Class V respectively, with higher scores indicating a poorer prognosis. Demographic information including sex, race, height, weight, body mass index (BMI), and presence of comorbid conditions (i.e. diabetes mellitus or cardiovascular disease) prior to admission were also collected. Presence of cardiovascular disease was defined as the patient having a history of any of the following conditions: hypertension, angina, myocardial infarction, or congestive heart failure.

### Data analysis

Data were analyzed using the Excel statistical package Analyse-it 5.68 (Leeds, UK). To assess the primary outcome Spearman correlations were conducted to assess continuous variables such as cumulative corticosteroid dose, BMI, weight, and patient acuity as assessed by both the SAPS II and BAP-65’s correlation to average hospitalization blood glucose level. All variables that showed a significant correlation were included in a multiple linear regression analysis. Based on biologic plausibility, the absence or presence of a history of diabetes was also added to the regression analysis utilizing dummy variables of zero and one respectively. Correlations were determined to be significant if the alpha ≤ 0.05. A similar process was conducted for the length of stay analysis.

Patients were eligible for the length of stay analysis if they were discharged from the hospital as medically stable (i.e., did not leave against medical advice or experience mortality) and received systemic corticosteroids within 24 hours of presentation to the emergency department. It was postulated that patients who had their initial systemic corticosteroid dose administered beyond this timeframe may have been admitted for a different primary problem with the AECOPD later recognized as a secondary problem during admission; thus, this barrier to the length of stay analysis was added.

For nominal secondary outcomes, cumulative corticosteroid doses were assessed for normality using the Shapiro-Wilk test. Patient groups were sub-divided on the basis of whether they did or did not experience various adverse reactions that could be related to corticosteroid excess, 30-day readmission, or mortality. Cumulative corticosteroid dose was compared between groups that did and did not experience each corticosteroid-related adverse drug reaction using the Mann-Whitney U test. As insulin administration could have been a competing risk for the development of hypokalemia, the total amount of as-needed correctional or one-time subcutaneous insulin was collected between the two following time points: after the first dose of corticosteroids was provided and up to the point of discharge or 24 hours after the final dose of corticosteroids, whichever came first. Data distributions are presented as occurrences with percentages and medians with interquartile ranges (IQR).

## Results

**Fig. 1:**
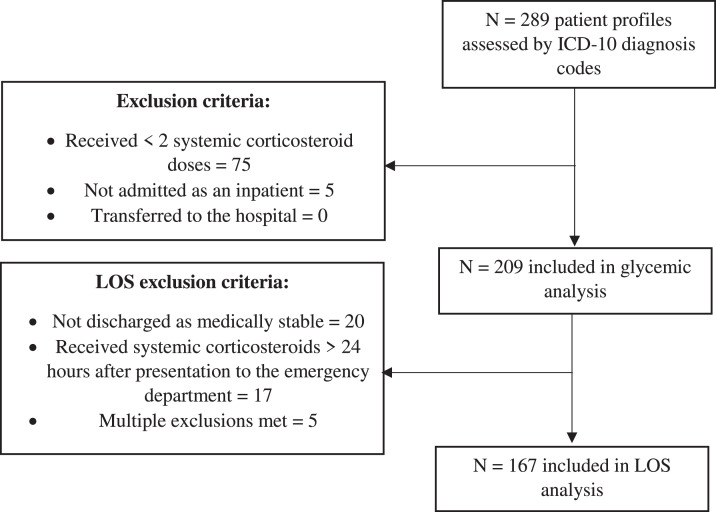
Length of stay analysis.

A total of 289 patients were assessed for inclusion from the ICD-10 report. Of those, 209 were included in the glycemic analysis. The primary reason for exclusion was administration of less than 2 systemic corticosteroid doses. Demographic information are described in Table 1. Of these, 167 patients were included in the length of stay analysis (Fig. 1). Notable baseline demographics include a 23.4% prevalence rate of diabetes, a median corticosteroid dose of 506 mg prednisone equivalents over the course of a hospitalization, a median hospital length of stay of approximately 3.9 days, and a calculated daily corticosteroid dose of 128 mg of prednisone equivalents per day.

**Table 1:** Demographic Information.

Variable	n (%) or Median (IQR)
Male sex	97 (46.4%)
History of diabetes	49 (23.4%)
Type 1 Diabetes	0 (0%)
Type 2 Diabetes	43 (20.6%)
Diabetes Type Unspecified	6 (2.9%)
Hemoglobin A1C of patients with noted history of diabetes	6.5 (6.1-7.3)
History of cardiovascular disease	180 (86.1%)
Diagnosis of pneumonia	61 (29.2%)
Required intensive care unit admission during hospitalization	65 (31.1%)
Race - White	188 (90.0%)
Ethnicity - Not Hispanic/Latino	188 (90.0%)
Age	77 (69-84)
BMI	26.5 (22.6-31.2)
Hospital LOS (days)	3.9 (2.7-6.8)
Corticosteroid Treatment Information	
Use of methylprednisolone as initial drug (excluding initial boluses)	188 (90.9%)
Initial dosing interval (hours, excluding initial boluses)	8 (6-8)
Initial dose amount (prednisone mg equivalents, excluding initial boluses)	50 (50-50)
Length of corticosteroid treatment (days)	4 (3-6)
Cumulative corticosteroid dose (prednisone milligram equivalents)	506 (307-776)
Average hospitalization BG (mg/dL, mmol/L)	133 (120-154), 7.38 (6.67-8.55)
BAP-65	3 (2-3)
SAPS II	29 (24-38)

BAP-65: clinical acuity marker considering presence of blood urea nitrogen above 25 mg/dL, altered mental status, pulse above 108 beats per minute, and age above 64 years; BG: Blood glucose; BMI: Body mass index; IQR: Interquartile range; LOS: Length of stay; SAPS II: Simplified acute physiology score II.

**Fig. 2:**
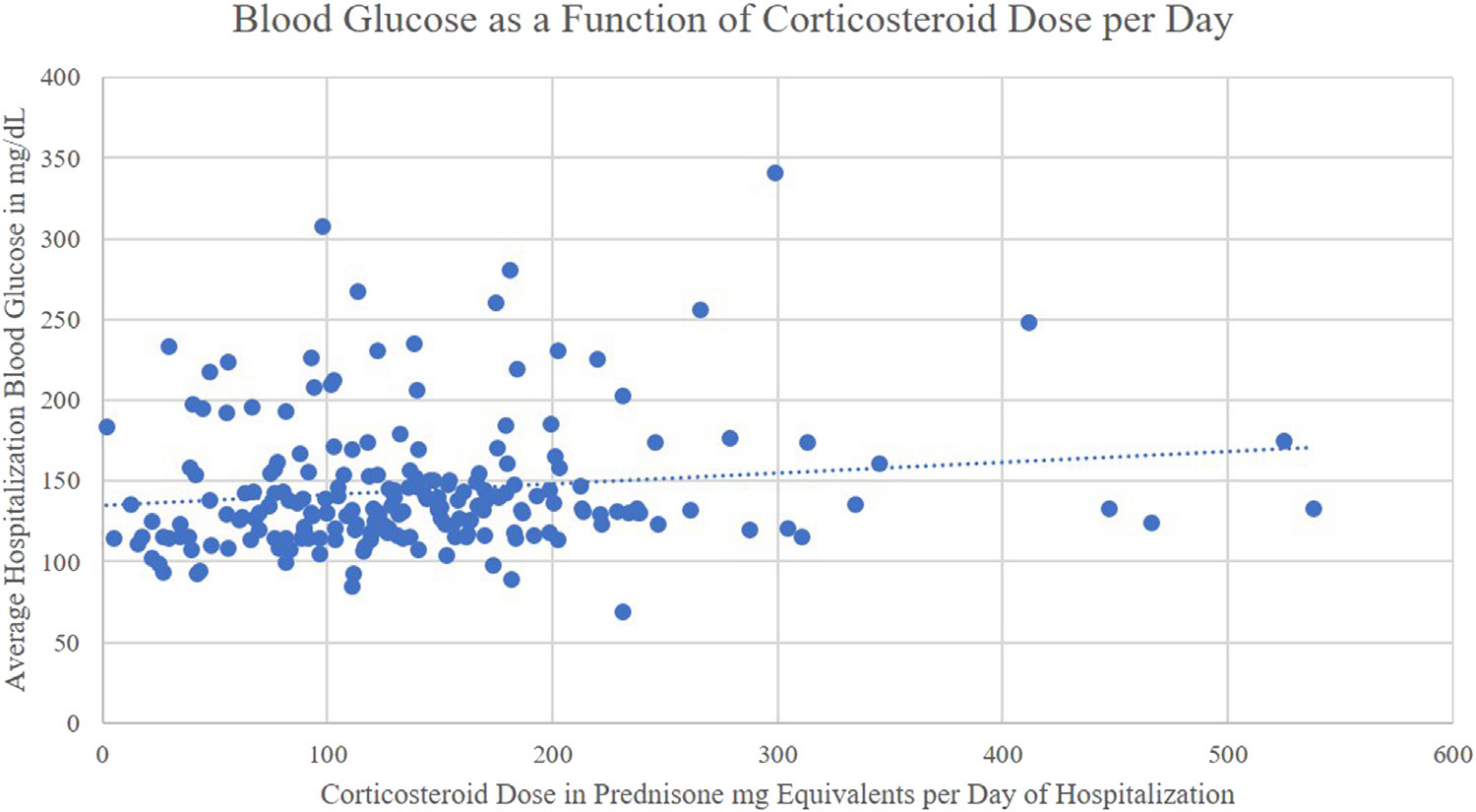
Blood glucose as a function of Corticosteroid dose per day.

The bivariate Spearman correlation analysis identified all continuous variables measured with the exception of age as being correlated with a higher average hospitalization blood glucose (Table 2). In the multiple regression analysis average daily corticosteroid dose, patient acuity as measured by the BAP-65, and the presence of diabetes were predictive of a higher average hospitalization blood glucose (Table 2). Using the slope estimate from this model predicts that every additional 100 mg of prednisone milligram equivalents administered per day raised a patient's average hospitalization blood glucose by 9.3 mg/dL (0.52 mmol/L, Fig. 2). Additionally, an increase in BAP-65 by one point and the presence of diabetes conferred an increase in average blood glucose by 6.85 mg/dL (0.38 mmol/L) and 46.7 mg/dL (2.59 mmol/L) respectively.

**Table 2:** Glycemic analysis.

Variable Compared to Average Hospitalization BG	Bivariate Spearman Correlation	Multiple Linear Regression
Cumulative Corticosteroid Dose	rs=0.248, p=0.0003	B=0.007, p=0.1773
Corticosteroid Dose per Day of Hospitalization	rs=0.179, p=0.0095	B=0.087, p=0.0028
BMI	rs=0.248, p=0.0003	B=-0.036, p=0.9626
Age	rs=-0.027, p=0.7011	-
Weight	rs=0.185, p=0.0072	B=-0.041, p=0.8726
BAP-65	rs=0.208, p=0.0024	B=6.85, p=0.0383
SAPS II	rs=0.213, p=0.0020	B=-0.096, p=0.7125
Noted history of diabetes	-	B=46.7, p<0.0001

BAP-65: clinical acuity marker considering presence of blood urea nitrogen above 25 mg/dL, altered mental status, pulse above 108 beats per minute, and age above 64 years; BG: Blood glucose; BMI: Body mass index; SAPS II: Simplified acute physiology score II.

**Fig. 3:**
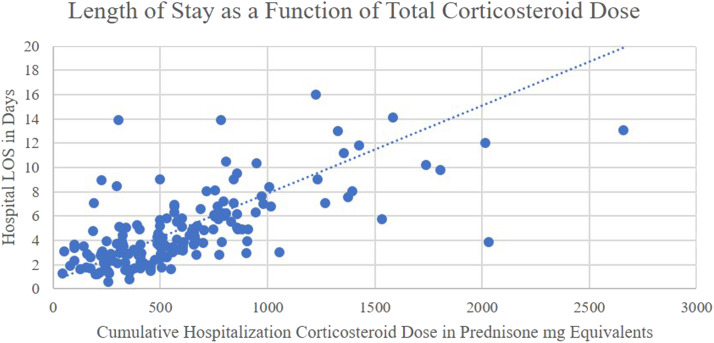
Length of stay as a function of total Corticosteroid dose.

In the length of stay analysis the two variables that retained statistical significance in the regression analysis were cumulative hospitalization corticosteroid dose and corticosteroid dose per day of hospitalization (Table 3). Using the slope estimate from this model predicted that every additional 100 mg of prednisone milligram equivalents administered during a hospitalization conferred an additional 0.9 days to the predicted length of stay (Fig. 3). Patient acuity markers and the presence of diabetes were not found to predict hospital length of stay (Table 3).

**Table 3:** LOS Analysis.

Variable Compared to LOS	Bivariate Spearman Correlation	Multiple Linear Regression
Cumulative Corticosteroid Dose	rs=0.679, p<0.0001	B=0.009, p<0.0001
Corticosteroid Dose per Day of Hospitalization	rs=-0.377, p<0.0001	B=-0.031, p<0.0001
Average Hospitalization BG	rs=0.023, p=0.7643	-
BMI	rs=0.149, p=0.0540	-
Age	rs=0.070, p=0.3689	-
Weight	rs=0.095, p=0.2234	-
BAP-65	rs=0.191, p=0.0134	B=-0.099, p=0.8162
SAPS II	rs=0.241, p=0.0017	B=-0.005, p=0.887
Noted history of diabetes	-	B=1.24, p=0.144

BAP-65: clinical acuity marker considering presence of blood urea nitrogen above 25 mg/dL, altered mental status, pulse above 108 beats per minute, and age above 64 years; BG: Blood glucose; BMI: Body mass index; LOS: Length of stay; SAPS II: Simplified acute physiology score II.

In regard to nominal secondary outcomes, patients who experienced in-hospital hypernatremia, hypokalemia, acute hyperglycemia, acute hypertension, and mortality received larger median cumulative corticosteroid doses than those patients who did not experience those events (Table 4). Median cumulative corticosteroid dose was not associated with 30-day hospital readmission (Table 4). There was no significant difference in correctional insulin dose provided between the patient populations that did and did not experience hypokalemia (0 units [0.0-4.0] vs. 0 units [0.0-3.2], p=0.659).

**Table 4:** Median Corticosteroid Dose in Patients that Did and Did Not Experience Complications During Hospitalization.

Medical Complication	Did experience complication during hospitalization- median CS dose (IQR)	Did not experience complication during hospitalization- median CS dose (IQR)	P-Value
Any Serum Sodium Value > 145 mEq/L (n=17)	1006.3 (633.3-1670.8)	500 (306.3-750.7)	0.0003
Any potassium value < 3.5 mEq/L (n=36)	822.5 (491.8.6-1343.6)	486.3 (300.0-700.0)	<0.0001
Any BG value >180 mg/dL (10 mmol/L, n=97)	643.8 (392.3-948.4)	456.3 (258.5-655.2)	0.0002
Any systolic blood pressure value > 180 mmHg (n=46)	750.6 (563.3-951.8)	456.3 (295.2-669.4)	<0.0001
Administration of an as-needed antihypertensive (n=42)	656.3 (404.6-900.7)	485.3 (301.0-723.3)	0.0065
In-hospital mortality (n=14)	787.5 (337.2-1609.4)	500 (306.3-756.0)	0.0286
Readmission within 30-days of hospital discharge (n=45)	566.3 (359.6-792.1)	456.6 (299.7-751.6)	0.1682

BG: Blood glucose, CS: Corticosteroid, IQR: Interquartile range.

## Discussion

Corticosteroids affect various physiologic processes and exert effects in the metabolic, respiratory, vascular, and other systems.^14^^,^^23^ In the context of an AECOPD, corticosteroids exert beneficial effects through optimizing spirometric parameters, improving gas exchange, and ultimately by improving clinical outcomes such as reducing the risk of treatment failure and shortening hospital length of stay.^9^^,^^24^, ^25^, ^26^, ^27^, ^28^ Proposed mechanisms underlying this clinical benefit include reducing systemic inflammation and maintaining beta-2 receptor sensitivity.^29^^,^^30^ In 2014 a Cochrane review estimated that, as compared to placebo, the provision of corticosteroids to patients experiencing an AECOPD reduced the risk of treatment failure within 30 days by approximately half (OR 0.48, 95%CI 0.35-0.67).^25^ International guidelines endorse routine use of a low dose and short duration of corticosteroids for the treatment of AECOPD. Current GOLD guidelines recommend a standard dose of prednisone 40 mg daily for 5 days.^1^

Although corticosteroid use in AECOPD can improve clinical outcomes, it may also increase the risk of hyperglycemia. Previous literature has illustrated use of corticosteroids in AECOPD nearly triples the risk of hyperglycemia.^25^ The mechanism of hyperglycemia is likely multifactorial and may involve induction of peripheral insulin resistance, decreased pancreatic insulin production, and other biochemical pathways.^13^

Previous literature has stated corticosteroids exert their hyperglycemic effect in a dose-dependent manner though this has been observed inconsistently at doses routinely used in the treatment of AECOPD.^17^^,^^18^^,^^31^ One retrospective study published in 2016 analyzed the dose-dependent effect of corticosteroids on risk of hyperglycemia in inpatients treated for AECOPD.^18^ Patients included in this trial were separated into low, moderate, or high dose tertiles based upon the corticosteroid dose they received during their second day of admission. The authors of this study noted a numerical increase in the risk of steroid-induced hyperglycemia moving from the low to moderate to high dose tertile, though this difference was not shown to be statistically significant. Additionally, a retrospective study published in 2018 divided inpatients treated for AECOPD in to standard and high dose corticosteroid groups based upon whether the patient received less than or equal to 200 milligrams of prednisone during their hospitalization or a dose greater than that.^17^ No difference was detected with regard to development of a new blood glucose elevation, defined as a fasting blood glucose ≥ 140 mg/dL (7.77 mmol/L) or any blood glucose ≥ 180 mg/dL (10 mmol/L), between the standard and high dose groups (79.5% vs. 78.8% respectively, P=0.9). The results from our study provide context and add additional specificity on to these previous reports. We identified that increasing doses of corticosteroids used commonly for AECOPD were correlated with increasing average hospitalization blood glucose concentrations in a linear manner.

There is a growing body of evidence that commonly used corticosteroid doses exceeding usual guideline recommendations are associated with a protracted hospital length of stay. The aforementioned 2018 study identified that patients in the high dose corticosteroid group had a longer hospital length of stay than those in the standard dose group (4 days [2-6] vs. 3 days [2-4.5], P<0.001).^17^ Additionally, a recent meta-analysis compared clinical outcomes of patients treated for AECOPD.^32^ Studies were enrolled in the meta-analysis if a specific dose of corticosteroid was compared against placebo, with studies being stratified as utilizing low, medium, or high corticosteroid doses. The low dose group was pre-specified as being 40 milligrams of prednisone per day or less, akin to current international guideline recommendations. The investigators identified that specifically the low dose corticosteroid group had a shorter hospital length of stay compared to placebo whereas the medium and high dose groups did not (mean difference –1.21 [95% CI –2.24 to –0.19], P =0.02). These findings are consistent with what was identified in our study in that larger cumulative doses of corticosteroids were correlated with longer hospital lengths of stay. We hypothesize that this correlation may be due in part to the observation that patients in our analysis that developed adverse effects related to corticosteroid excess tended to receive larger doses of corticosteroids. Development of adverse effects such as the observed electrolyte abnormalities, hypertension, or unmeasured glycemic variation may have mandated additional treatment that prolonged the individual patient's hospitalization. A recent prospective study identified that in inpatients with community acquired pneumonia increases in glycemic variability assessed by a continuous glucose monitor were predictive of increased hospital LOS.^33^ The results of this and our study highlight the importance of international guideline recommendations to surveil for steroid-induced hyperglycemia 1-2 hours after lunch, particularly in patients receiving high doses of corticosteroids.^33^^,^^34^ This observation must be taken in context with the limitations of this study though. In our retrospective study we were unable to proactively control for patient acuity; thus, this may have acted as a confounder in this analysis though the SAPS-II and BAP-65 patient acuity markers did not retain significance as predictors of LOS in our multivariate analysis. Additionally, while we observed that a larger cumulative corticosteroid dose was predictive of a longer hospitalization, we also identified a counterintuitive pattern that a larger corticosteroid dose per day of hospitalization had an opposite effect, apparently shortening hospitalization. This was an unexpected finding that is contrary to many recent publications and are uncertain as to whether this represents a de facto correlation or an organic causation. We posit that this finding could be due to frequent utilization of an inpatient corticosteroid taper in patients with a protracted length of stay in our dataset. This would result in the noted observation wherein a longer course of therapy is used though a lower calculated daily corticosteroid dose is realized due to smaller doses being incorporated at the end of therapy. Further research utilizing a standardized corticosteroid dose for the totality of a patient's hospitalization would be helpful in clarifying this point.

The goal of pharmacotherapy in AECOPD is ultimately to facilitate a favorable clinical recovery and timely discharge from inpatient services.^1^ Recent retrospective data have shown that prescribing corticosteroids consistent with guideline recommendations may lead to an improvement in clinical outcomes.^12^^,^^31^ In a quasi-experimental pre-post study clinical outcomes of AECOPD patients were assessed before and after the implementation of an inpatient AECOPD pharmacotherapy order set. The order set provided pre-populated recommendations for bronchodilators, antibiotics, and a corticosteroid regimen equivalent to prednisone 40 mg daily. After the implementation of this order set the risk of new-onset hyperglycemia and hospital length of stay were reduced (hyperglycemia 79.1% vs. 49.3%, P<0.001; length of stay 4.3 days vs. 3.4 days, P=0.004). These findings were noted alongside a reduced cumulative hospitalization corticosteroid dose and equivalent patient acuity. There are relatively few prospective corticosteroid dose-finding studies in AECOPD though multiple are ongoing. One published dose finding study compared the utility of a dynamic daily corticosteroid dosing algorithm based on patient-specific factors against a static daily dose of prednisone 40 mg daily in inpatients with AECOPD.^35^ Patients were randomized to receive one of the aforementioned corticosteroid dosing treatment plans for 5 days unless treatment failure was observed, in which case they could receive additional corticosteroid. The mean corticosteroid dose used between the personalized and standard dose cohorts was 61.4 mg and 56.2 mg per day respectively. While no difference in hospital length of stay was observed between the two groups, the personalized corticosteroid dosing group had a lower rate of in-hospital and medium-term treatment failure (27.6% vs. 48.8%, P=0.001). This difference in outcomes was largely attributed to use of prednisone 60 mg earlier in therapy and, with no observed differences in hyperglycemia between the two groups, raises the question as to whether 60 mg of prednisone a day may be considered a more appropriate corticosteroid regimen for patients with AECOPD. While somewhat higher than the current GOLD dosing schemata, a 60 mg prednisone per day regimen represents a much lower average dose than what was appreciated in our study. It may be that the risks of therapy increase with diminishing therapeutic returns when an average corticosteroid dose is progressed from 60 mg prednisone per day to 128 mg prednisone per day, the calculated average dose in our study. Despite relatively prescriptive guidance from current guidelines regarding dosing of systemic corticosteroids significant variability in practice patterns exist in the literature and within our study.^11^ Adherence to evidence-based and guideline endorsed corticosteroid treatment regimens in AECOPD may result in improved patient-centric clinical outcomes and reduced incidence of corticosteroid-related adverse events.

Our study is not without limitations. This data was collected by manual data extraction by one investigator and the possibility of transcription errors cannot be completely excluded. The selection of morning fasting blood glucose as the measurement incorporated into our primary outcome allowed for the most consistent and reproducible measure of blood glucose given the study's retrospective design. The selection of this measure may be less sensitive than other measures of glycemic excursion though as corticosteroids exert a more profound effect on postprandial than fasting blood glucose levels.^13^ Additionally, confounders affecting glycemic control, hospital length of stay, and the development of prespecified adverse effects may be present that were not thoroughly accounted for in our data collection form, including the exact corticosteroid and dosing interval utilized throughout the course of each patient's hospitalization. Medication use other than corticosteroids or acute diseases such as certain antibiotics, antipsychotics, parenteral nutrition, or pancreatitis, may have dysregulated normal metabolic response to physiologic stress. The inclusion of patients with both the presence and absence of a history of diabetes could have skewed our results as patients with a history of diabetes may have been more sensitive to the hyperglycemic effects of corticosteroids. Lastly, physician-specific prescribing habits were not completely accounted for. Discharge criteria for an AECOPD are non-prescriptive and may vary between physicians. It is easy to imagine a scenario wherein a physician who is particularly risk averse may not only institute a longer hospitalization but also larger doses of corticosteroids as part of a personal standard of practice.

## Conclusion

In our study we observed that larger daily corticosteroid doses in patients with AECOPD were correlated with higher average hospitalization blood glucose readings and greater cumulative doses were correlated with longer hospital lengths of stay. This was found in conjunction with corticosteroid doses that were on average much higher than current international guideline recommendations. Corticosteroid stewardship or monitoring programs may improve outcomes in inpatients with AECOPD. Prospective dose-finding studies are needed to identify the optimal corticosteroid dose for AECOPD. Future prospective studies implementing standardized corticosteroid treatment regimens and blood glucose collection schedules may more precisely define the extent of hyperglycemia induced by corticosteroids in AECOPD.

## Financial Disclosures

All authors declare no financial interest in companies/entities that either sponsor clinical research and/or develop, manufacture, or sell medications, medical devices, or biologics.

## Funding statement

No funding was utilized for this study.

## CRediT authorship contribution statement

**Herman Joseph Johannesmeyer:** Conceptualization, Methodology, Investigation, Formal analysis, Writing – original draft, Writing – review & editing. **Kayvan Moussavi:** Conceptualization, Methodology, Writing – review & editing. **Kerry Anne Rambaran:** Formal analysis, Writing – review & editing. **Kristica Kolyouthapong:** Conceptualization, Writing – review & editing.

## Declaration of Competing Interest

All authors declare no financial conflicts of interest.
